# Predictors of pesticide levels in carpet dust collected from child care centers in Northern California, USA

**DOI:** 10.1038/s41370-022-00516-8

**Published:** 2023-01-04

**Authors:** Kimberly Hazard, Abbey Alkon, Robert B. Gunier, Rosemary Castorina, David Camann, Shraddha Quarderer, Asa Bradman

**Affiliations:** 1 School of Public Health, University of California, Berkeley, CA USA; 2 School of Nursing, University of California, San Francisco, CA USA; 3 Southwest Research Institute, San Antonio, TX USA; 4 School of Social Sciences, Humanities and Arts, University of California, Merced, CA USA

**Keywords:** Child Exposure/Health, Children’s Health, Geospatial Analyses, Pesticides, Vulnerable Populations, Empirical/Statistical Models

## Abstract

**Background:**

Young children may be exposed to pesticides in child care centers, but little is known about determinants of pesticide contamination in these environments.

**Objective:**

Characterize pesticide contamination in early care and education (ECE) centers and identify predictors of pesticide concentrations and loading in dust collected from classroom carpets.

**Methods:**

Carpet dust samples were collected from 51 licensed child care centers in Northern California and analyzed for 14 structural and agricultural pesticides. Program characteristics were collected through administration of director interviews and observational surveys, including an integrated pest management (IPM) inspection. Pesticide use information for the prior year was obtained from the California Department of Pesticide Regulation to characterize structural applications and nearby agricultural pesticide use.

**Results:**

The most frequently detected pesticides were cis-permethrin (98%), trans-permethrin (98%), bifenthrin (94%), fipronil (94%), and chlorpyrifos (88%). Higher bifenthrin levels were correlated with agricultural applications within 3 kilometers, and higher fipronil levels were correlated with professional pesticide applications in the prior year. In multivariable models, higher IPM Checklist scores were associated with lower loading of chlorpyrifos and permethrin. Placement of the sampled area carpet was also a predictor of chlorpyrifos loading. The strongest predictor of higher pesticide loading for the most frequently detected pesticides was location in California’s San Joaquin Valley.

**Significance:**

Our findings contribute to the growing understanding that pesticides are ubiquitous in children’s environments. Pesticide levels in carpet dust were associated with some factors that ECE directors may have control over, such as IPM practices, and others that are beyond their control, such as geographic location. IPM is an important tool that has the potential to reduce pesticide exposures in ECE environments, even for pesticides no longer in use.

**Impact:**

One million children in California under six years old attend child care programs where they may spend up to 40 h per week. Children are uniquely vulnerable to environmental contaminants; however early care settings are under researched in environmental health studies. Little is known about predictors of pesticide levels found in environmental samples from child care facilities. This study aims to identify behavioral and environmental determinants of pesticide contamination in California child care centers. Findings can empower child care providers and consumers and inform decision makers to reduce children’s exposures to pesticides and promote lifelong health.

## Introduction

The majority of young children in the United States (U.S.) spend time in out-of-home care settings, with many preschool-age children spending half of their waking weekday hours in early care and education (ECE) programs [1]. Chemical exposures in ECE environments are of particular concern because young children are uniquely vulnerable to their adverse effects during critical windows of rapid development [2]. Previous studies have reported on the presence of pesticides in ECE facilities [3–6]. Studies suggest that early-life exposure to pesticides, even at low levels, can have adverse health effects such as respiratory symptoms and decreased lung function [7, 8], and impacts on neurological and behavioral development [9]. A meta-analysis found that exposure to chronic, low-dose indoor residential insecticides during early childhood is associated with an increased risk of leukemia and lymphoma among young children and young adults [10]. Physiologically-based pharmacokinetic modeling provides evidence of age-related differences of pesticide metabolism and neurotoxic susceptibility [11, 12].

Pesticide contamination may result from nearby agricultural or structural use. More than one billion pounds (or over 450 million kilograms) of pesticides are applied in the U.S. annually, with nearly 90% used for agriculture [13]. A survey of 637 child care center directors in California found that 90% reported at least one pest problem and half of these center directors reported using pesticides to control pests, with 47% reporting the use of aerosolized pesticides, which pose greater risk of exposure than pesticides applied as baits or gels [14]. ECE programs may also hire professionals that apply pesticides in or outside the facility to manage pest problems. The First National Environmental Health Survey of Child Care Centers measured pesticides in indoor floor wipe samples, with chlorpyrifos, diazinon, cis-permethrin, and trans-permethrin detected in more than 67% of the centers. In smaller studies of ECE programs, detectable levels of pesticides were found in all dust samples from 13 centers in North Carolina [5] and 22 centers in Ohio [15]. However, the predictors of pesticide levels in ECE programs have not been quantitatively assessed.

Indoor dust is an important exposure pathway for young children because they spend more time close to and in direct contact with the ground and have greater hand-to-mouth activity [16]. Carpet dust is a good environmental medium for assessing long-term indoor exposure because pesticides and other contaminants collect in dust over years, where they are protected from degradation by sunlight, moisture, and microorganisms [17, 18]. Dust concentration and loading are complimentary measures of indoor contamination. Loading (amount of contaminant per unit of flooring sampled) is generally considered to be a better indicator of exposure [19] and is more sensitive to recent cleaning practices, while concentration is generally more indicative of sources of contamination [20, 21].

In California, the Department of Pesticide Regulation (DPR) has enacted policies intended to reduce children’s exposures to pesticides in public schools and licensed ECE centers (“schoolsites”) by limiting agricultural pesticide applications near schoolsites and encouraging adoption of low-risk pest management practices, known as integrated pest management (IPM). Within 0.25 miles (approximately 402 m) of a schoolsite, growers cannot apply certain pesticides on school days [22]. The California Healthy Schools Act is a right-to-know law that provides parents and staff with information about pesticide use at schoolsites [23]. Additionally, licensed pest management professionals (PMPs) are required to report pesticide applications at schoolsites.

In the present study, we examine behavioral and environmental determinants of pesticide concentrations and loadings in carpet dust collected from 51 ECE centers. We utilize baseline data from an ongoing study examining pesticide use and exposure in Northern California ECE centers. We hypothesize that proximity to agricultural pesticide applications, storing pesticides onsite, fewer IPM practices, older building age, having a PMP apply pesticides in the past year, placement of sampled area carpet on carpeted flooring, and fewer pests observed onsite are associated with higher pesticide concentrations and loading for frequently detected pesticides measured in carpet dust.

## Materials/subjects and methods

### Study population

Data for this analysis were collected as part of the University of California, San Francisco, Healthy Children & Environments Study (HCES), a randomized-control trial examining the impact of an IPM intervention for ECE centers on pesticide exposure and health risks. The present analysis uses baseline data collected from 51 ECE centers from four northern California counties during the first three years of the study (November 2017-January 2018, August 2018-November 2018, and September 2019-November 2019). Inclusion criteria for the four counties is described by Alkon et al. 2022 [24]. Briefly, the two San Francisco Bay Area and two San Joaquin Valley counties were matched on geography, demographics, and agricultural pesticide use. There is high agricultural pesticide use in the San Joaquin Valley counties compared to the more urban/suburban Bay Area counties [25]. The Institutional Review Board at the University of California, San Francisco approved all study activities, and written informed consent was obtained from all center directors.

### Observational checklist and interview data

We collected information about practices and facility characteristics during ECE director interviews and observational checklists. During the baseline assessment stage, study staff completed two inspection checklists in each ECE center: The Integrated Pest Management Checklist for Early Care and Education Programs (IPM Checklist) and the Health and Safety Checklist for Early Care and Education Programs, both used in previous ECE environment studies [26–29]. The IPM Checklist has shown construct, content, face, and criterion validity [27], as well as having predicted change in child care studies [29]. The IPM Checklist includes 73 items with 8 subscales (outdoor: garbage, exterior, play area; indoor: kitchen, bathrooms, play areas, storage, staff area). For each subscale pest problems (pest or evidence of pest observed) were recorded in each location. For this project, the IPM Checklist included additional items to identify use of doormats, flooring and carpet types in the classroom, and the pesticide products stored on-site, including product active ingredients and U.S. EPA pesticide registration numbers.

A child care health consultant, a health professional trained to provide health and safety information specific to ECE settings, was assigned to each ECE program and interviewed the director. The interview collected information about the director (e.g., education level, years of experience); center characteristics (e.g., facility age, maintenance information); the center’s pest problems, pesticide use practices, and IPM policies and practices; and cleaning products and routines.

### Dust sample collection

Dust samples were collected using a high-volume surface sampler, a specially designed vacuum cleaner that collects particles >5 micrometer (μm) in diameter [30] (HVS3; Cascade Sampling Systems, Bend, OR). Samples were collected from the carpet where children have circle time and/or nap time (often the carpet is used for both). All sites had a carpet or area carpet that was at least 1 square meter (m^2^) in area, the minimum area needed for carpet dust sampling.

Study staff used a standard protocol [31] for HVS3 preparation, sampling, and prevention of cross-contamination. The sampling train, vacuum wheels, and collection bottle were cleaned with soap and water, rinsed with distilled water, and washed with isopropyl alcohol between uses. The sample collector wore nitrile gloves and boot covers to sample from an area of 1–2 m^2^. The exact area sampled, weather conditions, and GPS coordinates were recorded at the time of the sample collection. Sealed Teflon collection bottles containing the dust samples were labeled with the collection date and sample identification number, stored in a −20 °C freezer, and shipped via overnight mail on dry ice to Southwest Research Institute (SwRI) (San Antonio, TX), where they were stored in freezers until analysis.

### Laboratory analysis

SwRI measured concentrations and loadings of 14 pesticides in the dust: bifenthrin, chlorfenapyr, chlorpyrifos, cyfluthrin, cypermethrin, dacthal, deltamethrin, diazinon, esfenvalerate, fipronil, lambda-cyhalothrin, permethrin (cis- and trans-), and piperonyl butoxide. These represent a mix of pesticides used regionally in agricultural and structural pest control that have been previously measured in California ECE centers, plus several newer-use pesticides such as fipronil and chlorfenapyr.

For each dust sample, the total dust mass was passed through a 150-μm stainless steel sieve and the fine dust was weighed. The aliquot of the sample’s fine dust mass removed for extraction was: 1.0 gram (g) if this mass exceeded 1.0 g, 0.5 g if 0.5–1.0 g, 0.2 g if 0.2–0.5 g, or the entire fine dust mass if <0.2 g. One duplicate and one matrix spike sample were prepared for every 20 samples in the extraction batch from additional aliquots of the sample with the largest fine dust mass. Each aliquot in the batch was spiked with three labeled extraction surrogates (diazinon-d10, 13C6-cis-permethrin, and p-terphenyl-d14) and Soxhlet-extracted with 200 mL of dichloromethane:hexane (1:1) for 18 h, and the extract concentrated to 1.0 milliliter. The entire extract was passed for cleanup through a florisil column and the eluent concentrated to a final volume of 1.0 milliliter in hexane for analysis by Gas Chromatography/Mass Spectrometry (GC/MS). One solvent blank was extracted with each extraction batch of dust samples.

Analysis for the 14 targets was performed using an Agilent 6890 N/5973 GC/MS in selected ion monitoring mode with a 30-meter x 0.25-millimeter ID x 0.25 µm film thickness Phenomenex ZB-Semi-volatiles GC column. The instrument was scanned to monitor 2 to 4 selected ions per analyte. Quantification was performed using chlorpyrifos-d10 and trans-permethrin-13C6 as internal standards. The percent relative standard deviation of the analytes was maintained within 30% during each initial seven-point standard calibration. The percent difference of each analyte in the mid-level standard was maintained within 40% of the initial calibration value during continuing calibrations. Pesticide concentrations were determined in nanogram per gram (ng/g) of dust and pesticide loadings were derived by multiplying the concentrations (ng/g-dust) by the dust loading (g-dust/m^2^). Detection limits for each target analyte are shown in Supplementary Table 1.

### California department of pesticide regulation pesticide use information

California Department of Pesticide Regulation (DPR) Pesticide Use Report (PUR) data were used for the geospatial analysis of facility proximity to agricultural pesticide applications and reported structural pesticide applications by PMPs at the schoolsite. PUR agricultural pesticide use data includes application date, pounds of active ingredient applied, pounds of product applied, crop treated, and location geocoded to one-square mile sections defined by the U.S. Public Land Survey System. As part of the Healthy Schools Act, PMPs must report certain pesticide applications made at schoolsites annually to DPR. The PUR data obtained for these school sites via Public Records Request included county, school name, address, product name, active ingredient, location, applicator, and date.

Indoor dust pesticide exposure studies often select a radius of 1–4 kilometer (km) around residences to assess associations with nearby agricultural pesticide use [32–35]. Harnly et al. (2009) found significant associations within ≈23 km^2^ around the home which corresponds to a radius of approximately 2.7 km [32], and Gunier et al. (2014) found concentrations and loadings of manganese in house dust related to agricultural applications of manganese fungicides within 3 km of the residence [35]. We estimated agricultural use for each pesticide of interest from 2015 to 2019 within a 3 km radius around each ECE center using GPS coordinates recorded at the time of the sample collection and ArcGIS (ESRI, Redlands, CA). At the time of the analysis, PUR data was publicly available on the California pesticide information portal (calpip.cdpr.ca.gov) for 2015–2018, and 2019 data was provided by DPR staff. We selected 365 days prior to the dust sample collection date to align with questions asked in the director interview (past 12 months) and the time period often correlated with pesticide dust concentrations [34]. The density of agricultural pesticide use was estimated using methods described by Nuckols et al. [36]. Briefly, for each pesticide, the total reported kilograms applied within the 365 days prior to the date of the dust sampling is weighted by the proportion of the area of the 3 km buffer around the ECE center that intersects with the Public Land Survey System section where the application occurred to determine pesticide use in kg/km^2^.

### Statistical analyses

We first calculated descriptive statistics for demographic characteristics, pesticide detection frequencies, and distributions of pesticide concentrations and loadings. Among the 14 pesticides measured, we conducted further analyses on those with detection frequencies over 75%: bifenthrin, chlorpyrifos, fipronil, and permethrin (cis- and trans-). The sum of the two concentrations for the isomers cis-permethrin and trans-permethrin were used as a ∑permethrin value for consistency with PUR records. Spearman’s correlation coefficients were computed for each pesticide concentration and loading (for samples with measurements below the detection limit, a value was imputed as the limit of detection divided by the square root of two (DL/√2), then all values were natural log transformed) and continuous predictors. Tobit multivariable regression models were developed for each natural log transformed pesticide analyte (both concentration and loading), setting the lower bound at the detection limit. Tobit regression is an unbiased approach for analyzing truncated data when a portion of the measurements are less than the limit of detection, resulting in left-truncated data [37]. The transformed pesticide concentrations or loadings were the dependent variables and the environmental characteristics and behavioral practices were the predictors, controlling for other variables. Statistical analyses were conducted with Stata 15 (StataCorp. 2017. *Stata Statistical Software: Release 15*. College Station, TX: StataCorp LLC).

Independent variables used in the multivariable models were density of agricultural use of the specific pesticide active ingredient within 3 km over the 12 months preceding the date of the dust sample (continuous, kg/km^2^), if an application of the active ingredient was reported to DPR by a PMP within 12 months preceding the date of the dust sample (binary), observation of a product containing the active ingredient during baseline site visit (binary), IPM score based on the IPM checklist (total number of items answered “Yes” over the number of applicable questions), number of types of pests observed at site visit (categorized as none, one, or two or more), geographic region (San Joaquin Valley or Bay Area), and for loading models—placement of sampled carpet (categorized as area carpet on top of hard surface flooring, area carpet on top of carpeted flooring, or carpeted flooring without area rug). Building year (from director interview or county records) was considered in correlations, but excluded from multivariable models because building year was closely correlated with IPM Checklist score.

## Results

### ECE characteristics

Table 1 describes the ECE program and facility characteristics. Programs from the first three years of the study were distributed across the four participating counties. There was a mix of program types, including private, non-profit (*n* = 15); private, for-profit (*n* = 10); Head Start (*n* = 6); California State Preschool Programs (*n* = 5); and blended funding (*n* = 15). Programs ranged in size from 10 to 200 children, totaling 3327 children enrolled in the 51 participating ECE centers. Director experience in the ECE field ranged from 4 to 51 years.

**Table 1:** Child care center characteristics.

Program and director characteristics		ECE facility characteristics		Characteristics related to IPM/Healthy Schools Act	
Geographic region (*n* = 51)	n (%)	Building age (*n* = 51)		IPM Checklist score (*n* = 51)	
San Francisco Bay Area	25 (49%)	Mean (SD)	1988 (18)	Mean (SD)	73.15 (9.42)
San Joaquin Valley	26 (51%)	Min—Max	1940 - 2018	Min—Max	49.02–91.30
Program type (*n* = 51)		Doormat observed at main entrance (*n* = 50)	*n* (%)	Number of different pests observed (*n* = 51)	*n* (%)
Non-profit private	15 (29%)	No	3 (6%)	None	11 (21%)
For-profit private	10 (20%)	Yes	47 (94%)	1	28 (55%)
Head Start/Early Head Start	6 (12%)	Doormat observed at classroom exit to outdoors (*n* = 42)		2–4	12 (24%)
California State Preschool Program	5 (10%)	No	13 (31%)	Director knowledge of HSA (*n* = 51)	
Blended	15 (29%)	Yes	29 (69%)	Yes	29 (62%)
Program size (*n* = 51)		Circle-time carpet type (*n* = 50)		No	18 (38%)
10–49 children	21 (41%)	Low pile	42 (84%)	Director knowledge of IPM (*n* = 51)	
50–99 children	20 (39%)	Medium-high pile/plush	8 (16%)	Yes	26 (55%)
100–200 children	10 (20%)	Flooring under sampled carpet (*n* = 51)		No	21 (45%)
Director years of experience in child care (*n* = 51)		Laminate/hardwood/tile	29 (57%)	Center has IPM Coordinator (*n* = 51)	
4–19 years	21 (41%)	Carpet	18 (35%)	Yes	15 (29%)
20–35 years	26 (51%)	NA—Carpet sampled is base flooring	4 (8%)	No	25 (49%)
>36 years	4 (8%)	How often carpets are deep cleaned per year (*n* = 51)		Do not know	11 (22%)
Director education level (*n* = 51)		<1	1 (2%)	Center has written IPM policy (*n* = 51)	
Some college	4 (8%)	1–2	35 (73%)	Yes	14 (27%)
Associate’s	7 (14%)	3–12	12 (24%)	No	37 (73%)
Bachelor’s	29 (57%)	Agricultural application of pesticide within 3 km, past 12 months (PUR) (*n* = 51)		PMP or exterminator sprayed in past 12 months (director self-report) (*n* = 51)	
Master’s or higher	11 (21%)	Bifenthrin	29 (57%)	Yes	29 (57%)
		Chlorpyrifos	13 (25%)	No	6 (12%)
		Permethrin	10 (20%)	Do not know	16 (31%)
				Professional structural pesticide application in past 12 months (PUR) (*n* = 51)	
				Bifentrhin	6 (12%)
				Fipronil	2 (4%)
				Permethrin	2 (4%)
				Any active ingredient	12 (24%)
				Non-exempt pesticide product observed onsite (*n* = 51)	
				Bifentrhin	1 (2%)
				Permethrin	4 (8%)
				Any active ingredient	12 (24%)

*ECE* early care and education, *HSA* California Healthy Schools Act, *IPM* integrated pest management, *PMP* pest management professional, *PUR* California Pesticide Use Report.

A doormat was present at the entrance to the facility for 47 of 50 centers (94%). Among directors, 62% (*n* = 29) reported knowing about the Healthy Schools Act, 55% (*n* = 26) knew about IPM, 29% (*n* = 15) had an IPM coordinator (as required by the Healthy Schools Act), and 27% (*n* = 14) had a written IPM policy for the program. The average score on the IPM Checklist was 73 (SD = 9); scores among the San Joaquin Valley sites were about 10% higher than the Bay Area sites, and their facilities were newer on average. Seventy-eight percent of sites (*n* = 40) had a pest or evidence of pests observed by the researcher completing the IPM Checklist. Most sites had one type of pest observed, and the maximum was four different pests. The most common pests observed by study staff during the completion of the IPM Checklist were flies and spiders. The most common pests observed by directors over the past year were ants (49%), head lice (43%), flies (41%), and spiders (41%) (Supplementary Fig. 1). Over half of the directors (57%) stated that a PMP had applied pesticides within the previous year and 24% of programs (*n* = 12) had a “non-exempt” pesticide product onsite that requires reporting under the Healthy Schools Act.

### Pesticide levels in dust

Table 2 summarizes the distributions of all the pesticides analyzed in carpet dust. All ECE centers had at least one detectable pesticide in the carpet dust sample. The most frequently detected pesticides were: cis-permethrin (98%), trans-permethrin (98%), bifenthrin (94%), fipronil (94%), and chlorpyrifos (88%). Among these, chlorpyrifos had the lowest mean concentration and bifenthrin had the highest mean concentration. Piperonyl butoxide, cypermethrin, chlorfenapyr, deltamethrin, lambda-cyhalothrin, esfenvalerate, cyfluthrin, and dacthal were detected in 6–73% of samples at baseline with mean concentrations ranging from 0.2 ng/g (dacthal) to 893.6 ng/g (cypermethrin). Diazinon was not detected in any samples. The total number of detected pesticide analytes within each center ranged from three to twelve (Supplementary Figs. 2–4).

**Table 2:** Distribution of 14 pesticide concentrations and loading in dust (*n* = 51 centers).

Pesticide	Detection frequency	Concentration (ng/g)				Loading (ng/m2)				Physicochemical properties		Statewide pesticide use	
	Percent (n)	Mean (SD)	Median	95th	Max	Mean (SD)	Median	95th	Max	Half-life (Soil degredation, aerobic) (days)^a^	LogKow (Octanol-Water)^b^	Amount sold for use in CA, 2018, (kg)^c^	Amount applied for agricultural use in CA, 2018 (kg)^d^
cis-Permethrin	98% (50)	1467.7 (7087.1)	234.0	1870.0	50700.0	5035.5 (15994.5)	945.0	37600.0	104000.0	13*	6.47–7.15	261031*	42292*
trans-Permethrin	98% (50)	1508.4 (7878.9)	234.0	1670.0	56300.0	4994.7 (16853)	814.0	12700.0	115000.0		6.47–7.43		
Bifenthrin	94% (48)	1667.9 (9168.8)	151.0	2750.0	65600.0	4908.7 (19162.4)	476.0	20100.0	134000.0	87	6.19–8.15	519,812	118,955
Fipronil	94% (48)	166.6 (389.7)	53.9	412.0	2430.0	983.4 (1775.3)	199.0	4970.0	8870.0	142	3.71–6.64	86,966	0
Chlorpyrifos	88% (45)	5.7 (5.2)	4.3	18.8	25.1	35.4 (49.4)	17.9	154.0	239.0	386	4.66–4.96	268,305	272,151
Piperonyl butoxide	73% (37)	208.2 (372.6)	92.7	1160.0	1770.0	1382.5 (3865.4)	262.0	5280.0	26700.0	13	4.00–4.75	59,427	6507
Cypermethrin	55% (28)	893.6 (1901.8)	389.0	3390.0	12600.0	2968.9 (7455.9)	<DL	21000.0	38700.0	22	6.05–6.60	41,442	300
Chlorfenapyr	33% (17)	93.9 (268.8)	<DL	539.0	1670.0	412 (921.1)	<DL	2790.0	4570.0	1	4.76–5.51	1892	83
Deltamethrin	29% (15)	381.2 (2076.4)	<DL	1100.0	14800.0	6720.8 (44482.8)	<DL	5640.0	318000.0	58	6.12–6.20	3789	1
ƛ-Cyhalothrin	20% (10)	273.6 (1497.2)	<DL	236.0	10400.0	1263.8 (5822.2)	<DL	3630.0	36100.0	175	6.20–6.80	53,472	38,294
Esfenvalerate	16% (8)	111.9 (555.7)	<DL	324.0	3940.0	343 (1389.2)	<DL	1860.0	8080.0	67	6.20–6.76	17,084	18,114
Cyfluthrin	10% (5)	83.1 (296.1)	<DL	925.0	1480.0	834.4 (3895.1)	<DL	4780.0	25800.0	33	5.74–6.29	5250	4110
Dacthal	6% (3)	0.2 (0.8)	<DL	2.9	3.4	1.7 (9)	<DL	6.8	62.2	59	3.48–4.28	146,956	84345
Diazinon	0% (0)	—	—	—	—	—	—	—	—	9	3.80–3.86	31,613	15,161

*Data for permethrin.

^a^University of Hertfordshire, Pesticide Properties Database: https://sitem.herts.ac.uk/aeru/ppdb/.

^b^U.S. EPA CompTox Chemicals Dashboard, predicted range values: https://comptox.epa.gov/dashboard/.

^c^Reports of Pesticide Sold in California, CDPR: https://www.cdpr.ca.gov/docs/mill/nopdsold.htm.

^d^California Pesticide Information Portal Application (CalPIP), CDPR: https://calpip.cdpr.ca.gov/main.cfm.

### Pesticide use report (PUR) data

The amount of pesticide sold for use (agricultural and structural) in California as well as the amount reported in agricultural applications for all 14 pesticides are shown in Table 2. Some of the pesticides in this study are primarily or only used in agriculture, such as chlorpyrifos, whereas some are used in primarily non-agricultural applications, such as fipronil, and some pesticides are widely used for both agricultural and structural pest control, such as permethrin.

Most ECE centers were located within 3 km of an agricultural pesticide application in the year prior to the dust sample (Table 1). Detailed estimates of agricultural pesticide density are shown by region in Supplementary Table 2. In San Joaquin Valley counties, 24 of the 26 centers were within 3 km of at least one agricultural bifenthrin application that took place up to 365 days before the dust collection. The most heavily applied pesticide was chlorpyrifos, with a total of nearly 44 kg/km^2^ applied within 3 km of the child care centers, most of which took place in San Joaquin Valley counties.

There were 18 active ingredients applied at the ECE centers reported to DPR within 365 days preceding the dust sampling date (Supplementary Table 3). Among these active ingredients, bifenthrin applications accounted for the greatest proportion of applications (36%).

### Pesticide concentration correlations

Spearman’s rank correlation coefficients are shown in Table 3 for imputed, log-transformed pesticide levels and continuous predictor variables. Density of bifenthrin agricultural pesticide applications within 3 km was significantly correlated (*p* < 0.05) with higher bifenthrin dust concentrations (*r* = 0.38) and dust loadings (*r* = 0.44). Greater number of fipronil applications reported by a PMP was significantly correlated with higher fipronil dust concentrations and loadings (*r* = 0.30). Higher IPM Checklist scores were significantly correlated with lower chlorpyrifos concentrations (*r* = −0.28). Other correlation coefficients, including among the pesticide analytes and among the predictors can be found in Supplementary Table 4.

**Table 3:** Spearman Correlation between pesticide concentrations and loadings and predictors in continous form.

	Bifenthrin		Chlorpyrifos		Fipronil		∑Permethrin	
	Concentration	Loading	Concentration	Loading	Concentration	Loading	Concentration	Loading
Pesticide-specific variables								
Agricultural use of pesticide within 3 km, 1 year (kg/km^2^)								
Bifenthrin	0.38**	0.44**						
Chlorpyrifos			0.11	0.10				
Permethrin							0.20	0.06
Number of reported PMP applications of pesticide, 1 year								
Bifenthrin	0.13	0.00						
Fipronil					0.30*	0.30*		
Permethrin							−0.05	0.12
Pesticide products with Active Ingredient observed onsite								
Bifenthrin	0.12	−0.06						
Permethrin							0.23	0.02
Center-specific variables								
IPM Score (# IPM practices observed)	0.23	0.16	−0.28*	−0.21	−0.03	−0.01	−0.26	−0.17
Building year	0.10	0.04	−0.26	−0.18	0.02	0.01	−0.21	−0.10
Pests observed (# types of pests)	−0.10	−0.09	−0.04	−0.03	0.09	0.15	0.13	0.10

Values lower than detection limit were imputed as DL/√2, then all concentrations (ng/g) and loadings (ng/m^2^) were log-transformed.

*p < 0.05.

**p < 0.01.

### Multivariable models

**Fig. 1:**
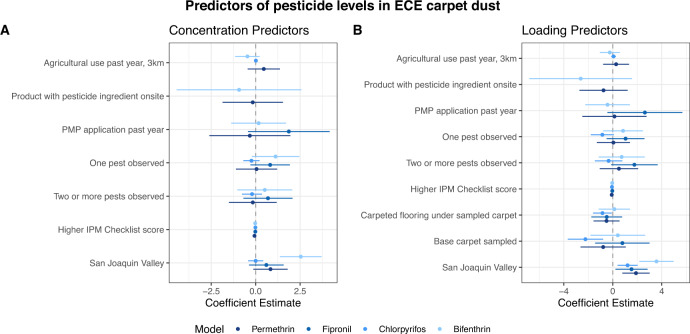
Coefficients and 95% confidence intervals for predictors of pesticide levels in ECE carpet dust. **A** Predictors modeled against four pesticide concentrations. **B** Predictors modeled against four pesticide loadings.

Results from the multivariable Tobit models for log-transformed pesticide concentrations and loading and predictor variables are shown in Table 4 and Fig. 1. We converted regression coefficients into percent change for the predictors (%Δ = (exp(β)-1)*100), also shown in Table 4. Location in the San Joaquin Valley was a significant predictor for higher concentrations of bifenthrin (1,166% (95% CI: 274%, 4,185%)) and bifenthrin loading (3,457% (95% CI: 733%, 15,086%)), chlorpyrifos loading (236% (95% CI: 43%, 691%)), fipronil loading (362% (95% CI: 20%, 1,682%)), and ∑permethrin loading (567% (95% CI: 112%, 2,001%)). Lower chlorpyrifos loading was associated with placement of the sampled carpet on carpeted flooring (−57% (95% CI: −81%, −5%)) and sampled base carpeting (−89% (95% CI: −98%, −50%)), compared to the referent placement of area carpet on hard-surface flooring. Higher scoring on the IPM Checklist was associated with lower permethrin dust loading (−8% (95% CI: −14%, −1%)) and lower chlorpyrifos dust loading (−6% (95% CI: −10%, −2%)).

**Table 4:** Multivariable Tobit model results for pesticide dust concentrations or loadings and predictors.

	Concentration (ng/g)				Loading (ng/m2)			
Pesticide	Predictor	β (95% CI)	Percent change (95% CI)	Pseudo R2	Predictor	β (95% CI)	Percent change (95% CI)	Pseudo R2
Bifenthrin				0.08				0.10
	Density of Ag Use (kg/km 2)	−0.46 (−1.18, 0.25)	−37% (−69%, 29%)		Density of Ag Use (kg/km 2)	−0.24 (−1.09, 0.6)	−21% (−66%, 83%)	
	PMP reported application in past year	0.17 (−1.42, 1.75)	18% (−76%, 478%)		PMP reported application in past year	−0.43 (−2.3, 1.45)	−35% (−90%, 325%)	
	Active ingredient observed onsite	−0.93 (−4.54, 2.68)	−61% (−99%, 1356%)		Active ingredient observed onsite	−2.62 (−6.94, 1.71)	−93% (−100%, 450%)	
	IPM Checklist score	−0.03 (−0.09, 0.04)	−2% (−8%, 4%)		IPM Checklist score	−0.05 (−0.12, 0.03)	−4% (−12%, 3%)	
	1 pest observed vs. no pests observed	1.12 (−0.26, 2.5)	206% (−23%, 1117%)		1 pest observed vs. no pests observed	0.84 (−0.85, 2.53)	132% (−57%, 1153%)	
	2+ pests observed vs. no pests observed	0.51 (−1.08, 2.11)	67% (−66%, 723%)		2+ pests observed vs. no pests observed	0.73 (−1.22, 2.67)	107% (−70%, 1350%)	
	Region: San Joaquin Valley	2.54 (1.32, 3.76)	1166% (274%, 4185%)		Region: San Joaquin Valley	3.57 (2.12, 5.02)	3457% (733%, 15086%)	
					Area sampled: Area rug on carpeted floor	0.13 (−1.19, 1.45)	14% (−70%, 327%)	
					Area sampled: Carpeted base flooring	0.41 (−1.89, 2.72)	51% (−85%, 1412%)	
Chlorpyrifos				0.08				0.15
	Density of Ag Use (kg/km 2)	0.05 (−0.07, 0.18)	5% (−7%, 20%)		Density of Ag Use (kg/km 2)	0.06 (−0.13, 0.26)	6% (−12%, 29%)	
	IPM Checklist score	−0.04 (−0.07, −0.01)	−4% (−7%, −1%)		IPM Checklist score	−0.06 (−0.11, −0.02)	−6% (−10%, −2%)	
	1 pest observed vs. no pests observed	−0.56 (−1.17, 0.05)	−43% (−69%, 5%)		1 pest observed vs. no pests observed	−0.85 (−1.82, 0.12)	−57% (−84%, 13%)	
	2+ pests observed vs. no pests observed	−0.5 (−1.23, 0.23)	−39% (−71%, 26%)		2+ pests observed vs. no pests observed	−0.34 (−1.5, 0.82)	−29% (−78%, 128%)	
	Region: San Joaquin Valley	0.26 (−0.29, 0.81)	30% (−25%, 124%)		Region: San Joaquin Valley	1.21 (0.36, 2.07)	236% (43%, 691%)	
					Area sampled: Area rug on carpeted floor	−0.84 (−1.64, −0.05)	−57% (−81%, −5%)	
					Area sampled: Carpeted base flooring	−2.23 (−3.77, −0.69)	−89% (−98%, −50%)	
Fipronil				0.03				0.06
	PMP reported application in past year	1.87 (−0.49, 4.23)	547% (−39%, 6748%)		PMP reported application in past year	2.62 (−0.52, 5.77)	1279% (−41%, 31976%)	
	IPM Checklist score	−0.02 (−0.07, 0.04)	−2% (−7%, 4%)		IPM Checklist score	−0.04 (−0.11, 0.04)	−4% (−11%, 4%)	
	1 pest observed vs. no pests observed	0.82 (−0.33, 1.96)	126% (−28%, 609%)		1 pest observed vs. no pests observed	1.05 (−0.54, 2.64)	185% (−42%, 1299%)	
	2+ pests observed vs. no pests observed	0.69 (−0.73, 2.12)	100% (−52%, 729%)		2+ pests observed vs. no pests observed	1.77 (−0.19, 3.72)	485% (−17%, 4039%)	
	Region: San Joaquin Valley	0.6 (−0.4, 1.6)	83% (−33%, 395%)		Region: San Joaquin Valley	1.53 (0.18, 2.88)	362% (20%, 1682%)	
					Area sampled: Area rug on carpeted floor	−0.49 (−1.79, 0.8)	−39% (−83%, 123%)	
					Area sampled: Carpeted base flooring	0.79 (−1.5, 3.07)	119% (−78%, 2051%)	
∑Permethrin				0.03				0.07
	Density of Ag Use (kg/km 2)	0.46 (−0.48, 1.39)	58% (−38%, 303%)		Density of Ag Use (kg/km 2)	0.27 (−0.81, 1.36)	32% (−56%, 290%)	
	PMP reported application in past year	−0.33 (−2.67, 2.02)	−28% (−93%, 652%)		PMP reported application in past year	0.14 (−2.56, 2.84)	15% (−92%, 1615%)	
	Active ingredient observed onsite	−0.16 (−1.91, 1.59)	−15% (−85%, 388%)		Active ingredient observed onsite	−0.75 (−2.78, 1.27)	−53% (−94%, 257%)	
	IPM Checklist score	−0.06 (−0.12, 0)	−6% (−12%, 0%)		IPM Checklist score	−0.08 (−0.15, −0.01)	−8% (−14%, −1%)	
	1 pest observed vs. no pests observed	0.05 (−1.14, 1.24)	5% (−68%, 246%)		1 pest observed vs. no pests observed	0.05 (−1.34, 1.44)	5% (−74%, 324%)	
	2+ pests observed vs. no pests observed	−0.16 (−1.54, 1.22)	−15% (−79%, 239%)		2+ pests observed vs. no pests observed	0.5 (−1.11, 2.12)	65% (−67%, 734%)	
	Region: San Joaquin Valley	0.84 (−0.16, 1.83)	131% (−15%, 524%)		Region: San Joaquin Valley	1.9 (0.75, 3.05)	567% (112%, 2001%)	
					Area sampled: Area rug on carpeted floor	−0.51 (−1.6, 0.58)	−40% (−80%, 79%)	
					Area sampled: Carpeted base flooring	−0.78 (−2.7, 1.13)	−54% (−93%, 210%)

## Discussion

Our results indicated that, for specific pesticides, geographic region, proximity to agricultural pesticide applications, applications of structural pesticides, fewer IPM practices, and placement of sampled carpet on hard surface flooring were predictors of higher pesticide levels in carpet dust Northern California ECE centers. The strongest predictor of higher pesticide loading for all the most frequently detected pesticides was location in the San Joaquin Valley. Correlations were strongest for bifenthrin levels and agricultural bifenthrin use within 3 km of the ECE center in the past year; fipronil levels and PMP applications of fipronil at the ECE center; and lower chlorpyrifos levels with better IPM practices. Overall, we saw stronger associations between our selected predictors with the pesticide loading than with concentration. We did not find associations between observed pesticide products stored onsite, pests observed, or age of the facility. Our findings contribute to the growing knowledge that pesticides are ubiquitous in the environments in which California’s youngest and most vulnerable populations are cared for.

**Fig. 2:**
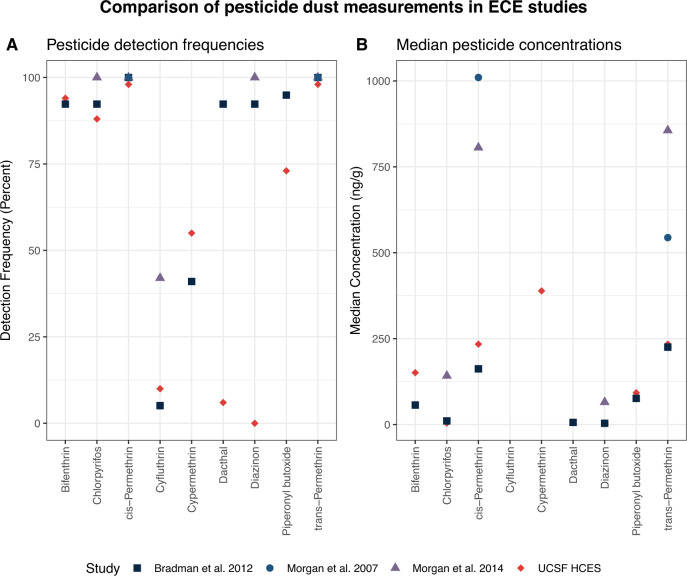
Comparison of pesticide measurements among studies with dust samples from early care and education (ECE) programs. **A** Comparison of pesticide detection frequencies among four ECE studies. **B** Comparison of median pesticide concentrations among four ECE studies. UCSF HCES = University of California, San Francisco, Healthy Children and Environments Study.

The distribution of pesticides in our study were consistent with that of a study in California child care centers reported by Bradman et al. 2012 [6] which examined 10 of the same target analytes from samples collected in 2010 and 2011 (Fig. 2 and Supplementary Table 5). Overall, the detection frequencies were similar aside from diazinon and dacthal, which were lower in our study. Chlorpyrifos was found at lower concentrations in the current study than other ECE studies. This is consistent with the declining use of organophosphate pesticides after a voluntary phase-out for indoor uses of chlorpyrifos and diazinon between 2001 and 2004 [38], and declining agricultural use in California which dropped more than 50% since 2005, and all sales of chlorpyrifos ceased in 2020 [39]. Median concentrations were similar for permethrin and piperonyl butoxide, and higher for bifenthrin and cypermethrin in our study, which may reflect increasing use of pyrethroids for pest control. In a study of 13 ECE programs in North Carolina, cis- and trans-permethrin were also highly frequently detected in dust samples [5] (Fig. 2). To our knowledge, this is the first study to measure fipronil and chlorfenapyr in carpet dust from ECE programs, two relatively new insecticides that are increasing in popularity [40, 41], and were detected in 94% and 33% of our samples, respectively.

Predictors of pesticide dust contamination were generally consistent with predictors of pesticide concentrations in passive sampling silicone wristbands worn by preschool-age children in the same study population [24]. Having a professional exterminator used in last 6 months at home was associated with higher levels of bifenthrin in wristbands worn by children. Pounds of agricultural pesticide use at the county-level was associated with cypermethrin, fipronil, and permethrin levels in the child wristbands, which is consistent with our findings of strong associations between region and higher concentration of bifenthrin and all pesticide dust loadings. In the silicone wristbands, having no pests observed at the ECE facility was associated with higher levels of bifenthrin, fipronil, and trans-permethrin, but we found no association between pests observed and pesticide levels in dust.

Most of what is known about determinants of non-dietary exposure to pesticides comes from studies in residential environments and suggest that both nearby agricultural pesticide use and individual behaviors are associated with pesticide exposures. A systematic review of non-dietary exposure to agricultural pesticides identified key determinants of exposure, including behaviors like housekeeping practices, and spatial indicators like proximity to fields and total amounts of pesticides applied near homes [42]. Harley et al. (2019) reported that living within 100 m of active agricultural fields, having carpeting in the home, and having an exterminator treat the home in the past six months were associated with higher odds of detecting certain pesticides in silicone wristbands, while concentrations were lower for participants who cleaned their homes daily and had doormats in the entryway of their home [43]. Several studies have reported that closer proximity to agricultural pesticide applications is associated with higher concentrations and loadings of pesticides in residential carpet dust [32, 34, 44, 45].

The correlation between bifenthrin levels and agricultural use within 3 km in the present study is consistent with associations in residential settings. We did not find an association with chlorpyrifos levels and agricultural use, despite chlorpyrifos only having agricultural uses in California during the study period. The half-life of chlorpyrifos can exceed one year (see Table 2), therefore we may need to examine associations with applications made within two or more years prior to the dust sample. To our knowledge, this is the first examination of agricultural proximity to child care centers and pesticide exposures. Further investigation is needed to determine if California’s regulatory buffer of <500 meters around schoolsites will sufficiently reduce exposure to agricultural pesticides.

We were not able to thoroughly examine other known predictors of residential pesticide contamination [42, 43]. For example, we were not able to examine heterogenous patterns for doormats, carpet deep cleaning, or daily cleaning practices. Most ECE programs had their carpets deep cleaned (steam cleaned, shampooed, sent out to cleaner, or other wet cleaning method) at least once per year, only three programs did not have a doormat at their entrance, and routine cleaning, sanitizing, and disinfecting is required by California child care licensing. We did not find any correlation between frequency of deep carpet cleaning and pesticide levels in preliminary analyses. It is notable that there are still measurable concentrations of at least one pesticide in dust from all ECE centers in this study, despite many common practices that should reduce contamination.

We found lower levels of permethrin and chlorpyrifos associated with higher scores on the IPM Checklist. Considering that chlorpyrifos has not been used indoors for more than two decades, this finding suggests that IPM practices may reduce exposure to legacy pesticides that persist in the indoor environment, in addition to preventing pest infestations and reducing the need for new pesticide applications. The IPM Checklist captures some information about building quality, doormats, ventilation, and cleaning practices, which may influence presence of persistent contaminants indoors.

Flooring type and presence of carpets are predictors of total indoor dust loading [46]. We found no difference in loading by the type of carpet sampled (low pile vs. medium and high pile). We hypothesized that pesticide levels would be lower in ECE centers with hard surface flooring types, however it appears that the placement of the sampled area carpet on laminate/hardwood/tile flooring in 29 of the 51 centers permitted ready entrainment of fine dust from the hard flooring with activity in the room and subsequent settling and collection on the sampled area carpet, producing the elevated chlorpyrifos and permethrin loading on these carpets. By contrast, less entrainment of fine dust may have occurred in the 18 centers where the sampled area carpet was placed on carpeted flooring and in the 4 centers where the carpet sampled was the base carpeting. This finding does not suggest that carpeted flooring is better than non-carpeted flooring for reducing exposure, but supports the notion that all carpets, particularly area carpets on which children come in close contact with, serve as reservoirs for indoor dust [47], and therefore should be targeted for frequent cleaning, and children’s hands should be washed after contact with carpets to reduce exposure. We were not able to determine the overall ratio of different flooring types in the classroom, and we relied on self-reported cleaning practices and frequencies. Placement of sampled carpets on different flooring types is a novel investigation in exposure assessment literature, and more research is needed.

It is of note that we found poor concordance between the director interview and data on PMP applications provided by DPR. There were instances when the ECE director reported that a PMP sprayed pesticides in the past year, but no PUR record was provided, and vice versa. For over 40% of centers (*n* = 21), the PUR data contradicted the self-reported data from the director interview. We used DPR data assuming it would be more accurate, but that is unconfirmed. A potential limitation of the PUR data is that it includes pesticide applications reported to DPR by licensed PMPs and does not include applications by unlicensed center staff; additionally, some ECE centers are located on a school campus, so applications may be reported for those schools and not shown for the childcare center. However, the PMP records include detailed information about application dates, location, and active ingredient(s). Overall, we found that using the PUR data returned stronger and more precise effect estimates compared to director reported information about PMP practices. Our findings suggest that self-report of PMP pesticide use is not as reliable as statewide PUR data, and that there may be an overall need for better communication between PMPs and ECE directors.

Limitations of this study include the relatively small number of baseline dust samples available from the first three years of HCES (sampling was curtailed due to COVID-19 restrictions) which limited our power to detect associations between pesticide levels and predictors. We enrolled a convenience sample of ECE programs and assessed exposure during a limited period (Fall to early Winter), therefore results are limited in generalizability. Data for certain predictors of pesticide levels were not collected or analyzed, such as measures of classroom ventilation, efficiency of vacuum used in classroom, or wind direction at time of agricultural pesticide applications. We also collected a single sample from one area of the classroom, rather than multiple samples throughout the center. Lastly, this analysis considers center-level predictors and single pesticide outcomes individually and does not account for chemical-specific characteristics such as vapor pressure or persistence, nor considers predictors of pesticide mixtures.

In conclusion, we found that pesticide levels in classroom carpet dust were associated with some factors that ECE directors may have control over (IPM practices and the use of a pest management professional) and others that are beyond their control (geographic location and proximity to agricultural pesticide applications). Children’s care environments are generally understudied, but are a critical point for intervention as chronic, low-level exposures in early childhood can influence lifelong health and development.

### Supplementary information

Supplementary Files

## Data Availability

De-identified data are available from the corresponding author on reasonable request.
