# Supplementary material for: Predictors of pesticide levels in carpet dust collected from child care centers in Northern California, USA
Source: J Expo Sci Environ Epidemiol. 2023 Jan 4;34(2):229–40. doi: 10.1038/s41370-022-00516-8 (PMC9811891; doi:10.1038/s41370-022-00516-8)
Supplement: Supplementary file 1 — Supplementary Files [file 41370_2022_516_MOESM1_ESM.pdf]

Supplementary Files

Predictors of pesticide levels in carpet dust collected from child care centers in Northern California, USA

Contents

Supplemental Figure 1. Director-reported pests observed, past year (director interview). 2

Supplemental Figure 2. Distribution of pesticide concentrations per dust sample (concentrations are log_{10}-transformed) 3

Supplemental Figure 3. Distribution of pesticide loading per dust sample (log_{10}-transformed) 4

Supplemental Figure 4. Total number of detected pesticides per dust sample (n=51). 5

Supplemental Table 1. Laboratory Analytical Data: Recovery of spiked pesticide amount in seven matrix spikes, and detection limits. 6

Supplemental Table 2. Estimated density of agricultural pesticide use by region, 3 km around center, 365 days prior to sampling... 7

Supplemental Table 3. Pest Management Professional (PMP) pesticide applications reported to DPR – at child care address, 365 days prior to sampling – by active ingredient.. 8

Supplemental Table 4. Correlations among pesticide concentrations and loadings, and among continuous predictors. 9

Supplemental Table 5. Comparison of pesticide measurements in early care and education (ECE) studies. Detection frequencies (DF%) and concentrations (ng/g). 10

Supplemental Figure 1. Director-reported pests observed, past year (director interview).

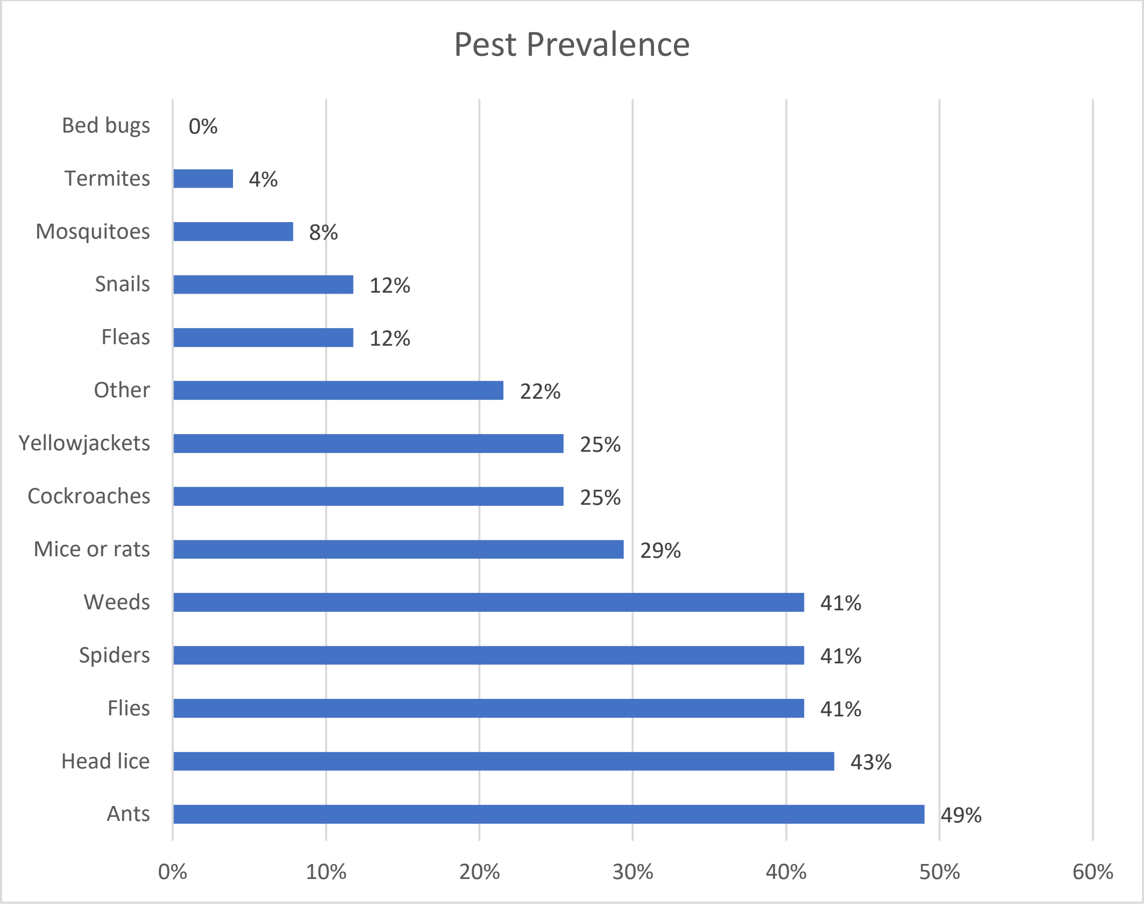

Supplemental Figure 2. Distribution of pesticide concentrations per dust sample (concentrations are log_{10}-transformed)

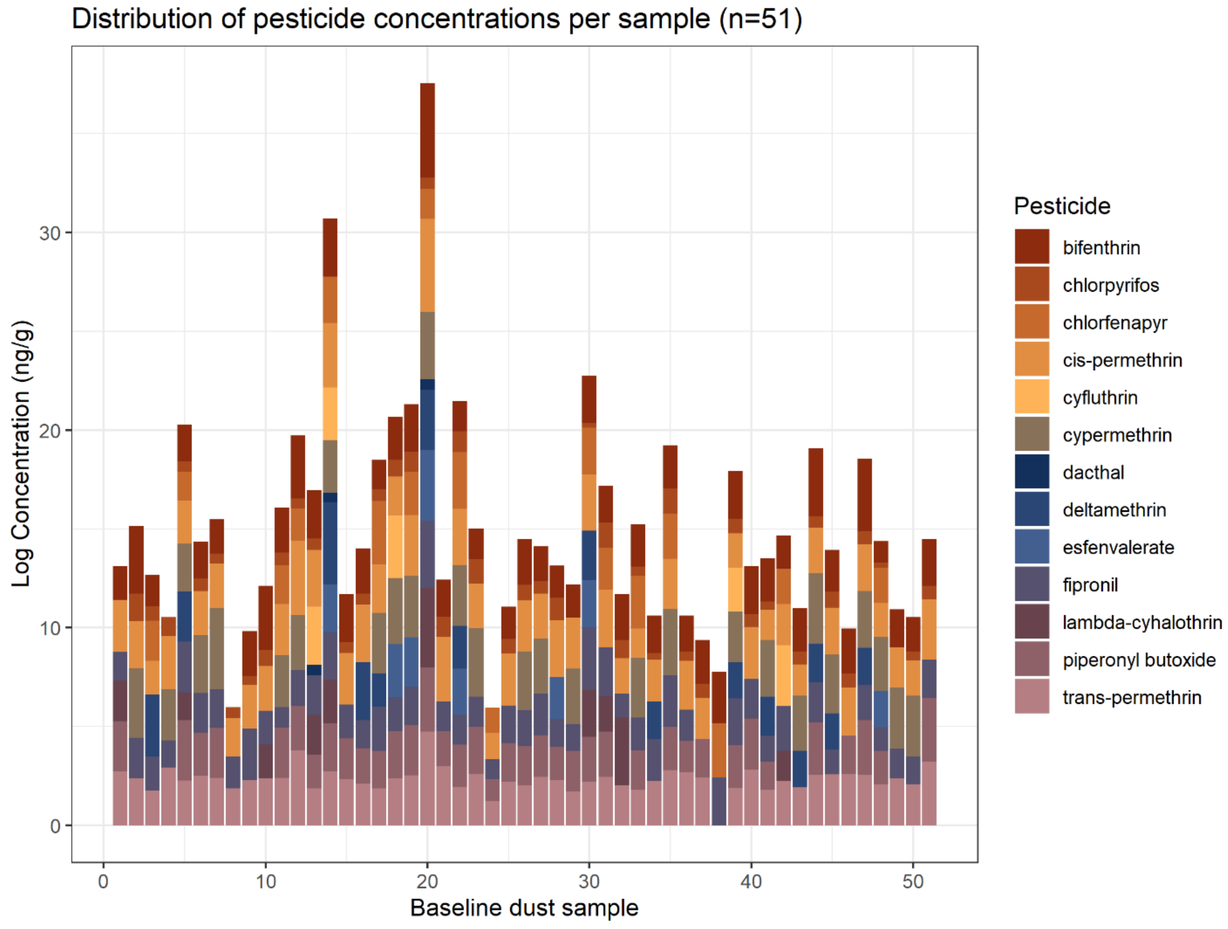

Supplemental Figure 3. Distribution of pesticide loading per dust sample (log_{10}-transformed)

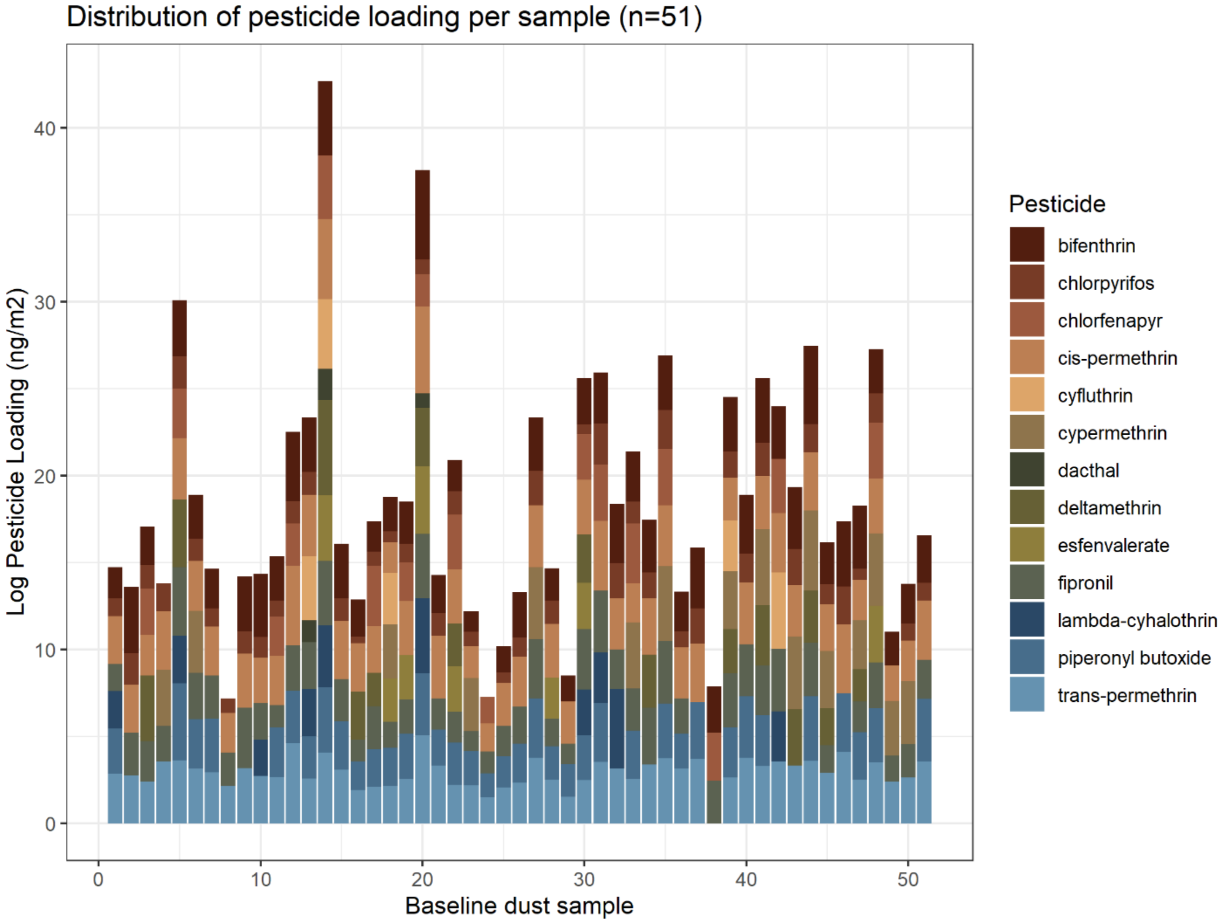

Supplemental Figure 4. Total number of detected pesticides per dust sample (n=51).

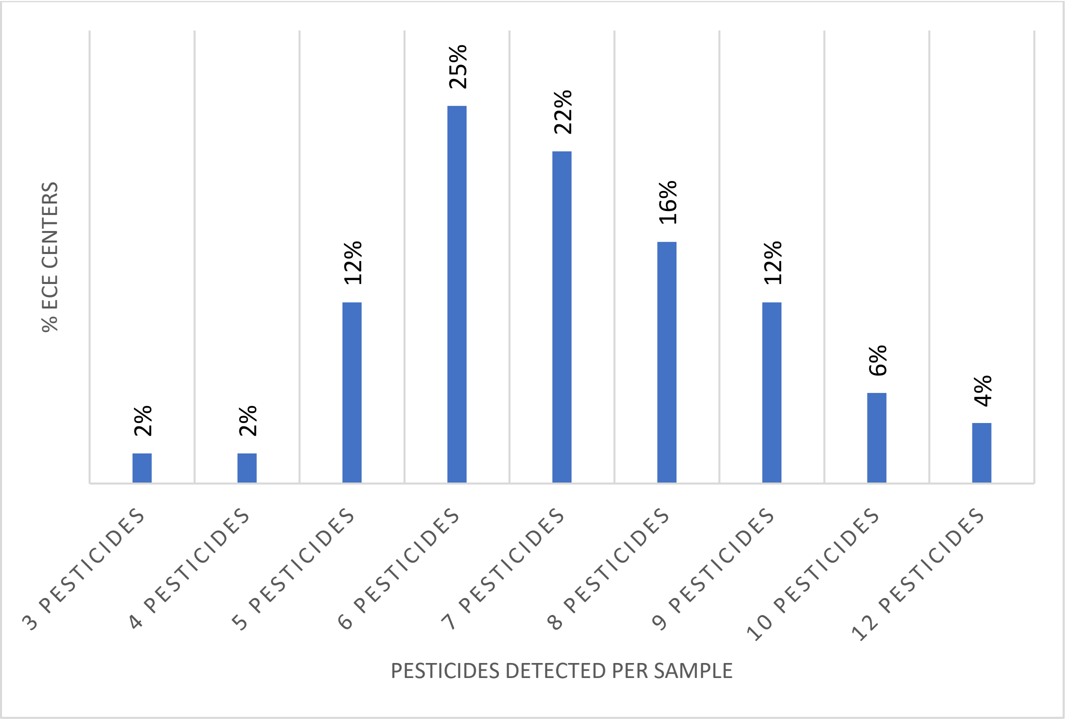

Supplemental Table 1. Laboratory Analytical Data: Recovery of spiked pesticide amount in seven matrix spikes, and detection limits.

Pesticide	Recovery (%)	Limit of Detection (ng/g)		
	Mean (SD)	Min	Median	95th percentile
**cis-Permethrin**	78 (15)	0.379	3.9	37.5
**trans-Permethrin**	88 (25)	1.52	12.6	121
**Bifenthrin**	97 (27)	1.26	1.66	16.3
**Fipronil**	91 (11)	2.53	3.31	16.5
**Chlorpyrifos**	92 (12)	1.26	1.65	3.26
**Piperonyl butoxide**	102 (27)	1.26	16.3	82.3
**Cypermethrin**	96 (73)	12.6	82.1	412
**Chlorfenapyr**	110 (39)	2.53	3.31	16.5
**Deltamethrin**	74 (17)	6.31	40.7	79.5
**λ-Cyhalothrin**	109 (33)	6.31	8.27	16.6
**Esfenvalerate**	92 (38)	6.31	41.1	79.5
**Cyfluthrin**	109 (46)	12.6	81.5	159
**Dacthal**	79 (5)	1.26	1.65	3.26
**Diazinon**	85 (7)	1.26	1.65	3.26

Measurement accuracy for each pesticide was assessed by the percent recovery of the spiked amount of each targeted pesticide in the seven matrix spike samples. Measurement precision was assessed by degree of agreement between the concentrations of each pesticide measured in the dust sample and its laboratory-split duplicate over the seven duplicate samples. Agreement was within a factor of two for 59 (95%) of the 62 pairs with detected concentrations in both the sample and its duplicate. Agreement was within 20% for 47 (76%) of these 62 duplicate pairs.

LODs were sometimes raised due to inseparable coeluting analytical interference compounds. We report the minimum, median and an upper percentile of the LODs for each target analyte across all of the baseline samples. Comparing the upper percentile and median to the minimum will indicate the prevalence of raised detection limits for each target pesticide.

Supplemental Table 2. Estimated density of agricultural pesticide use by region, 3 km around center, 365 days prior to sampling.

Pesticide	Region	# ECE Centers with application within 3 km, past year	Median (kg/km^2)*	Range (kg/km^2)*
**Bifenthrin**	SF Bay Area	5	1.79	0.57 - 2.21
	San Joaquin Valley	24	0.65	0.01 - 3.28
	Total	29	0.69	
**Chlorpyrifos**	SF Bay Area	2	4.60	4.03 - 5.16
	San Joaquin Valley	11	2.54	0.01 - 8.01
	Total	13	3.08	
**Permethrin**	SF Bay Area	2	1.18	1.17 - 1.19
	San Joaquin Valley	8	0.24	0.001 - 2.72
	Total	10	0.40	

*Among non-zero values

Supplemental Table 3. Pest Management Professional (PMP) pesticide applications reported to DPR – at child care address, 365 days prior to sampling – by active ingredient.

**Active Ingredient**	Number of applications reported **n (%)**
**Alpha-(para-nonylphenyl)-omega-hydrox..**	1 (1%)
**Bifenthrin**	29 (36%)
**Bromadiolone**	3 (4%)
**Chlorfenapyr**	1 (1%)
**Cyfluthrin**	2 (2%)
**Cypermethrin**	3 (4%)
**Deltamethrin**	16 (20%)
**Dinotefuran**	1 (1%)
**Edta, tetrasodium salt**	1 (1%)
**Esfenvalerate**	1 (1%)
**Fipronil**	3 (4%)
**Hydroprene**	2 (2%)
**Indoxacarb**	3 (4%)
**Permethrin**	2 (2%)
**Piperonyl butoxide**	5 (6%)
**Prallethrin**	1 (1%)
**Pyrethrins**	4 (5%)
**S-methoprene**	3 (4%)
**Total**	81 (100%)

Supplemental Table 4. Correlations among pesticide concentrations and loadings, and among continuous predictors.

Correlation among pesticide levels								
	Bifenthrin (BIF)		Chlorpyrifos (CPF)		Fipronil (FIP)		Permethrin (PERM)	
	Concentration	Loading	Concentration	Loading	Concentration	Loading	Concentration	Loading
BIF Concentration	1.00							
BIF Loading	0.78	1.00						
CPF Concentration	0.00	-0.09	1.00					
CPF Loading	0.05	0.44	0.44	1.00				
FIP Concentration	0.32	0.30	-0.10	-0.07	1.00			
FIP Loading	0.24	0.48	-0.27	0.21	0.81	1.00		
PERM Concentration	0.26	0.06	0.37	-0.01	0.16	0.05	1.00	
PERM Loading	0.27	0.61	0.01	0.55	0.25	0.53	0.49	1.00

Correlation among continuous predictors											
	IPM score	Building year	Pests observed (# types of pests)	Density of BIF Ag Use (kg/km^2)	Density of CPF Ag Use (kg/km^2)	Density of PERM Ag Use (kg/km^2)	BIF applications by PMP	FIP applications by PMP	PERM applications by PMP	BIF product onsite	PERM product onsite
IPM average score	1.00										
Building year	0.49	1.00									
Pests observed	-0.36	-0.30	1.00								
Density of BIF Ag Use	0.28	0.19	-0.05	1.00							
Density of CPF Ag Use	0.15	0.24	-0.07	0.39	1.0						
Density of PERM Ag Use	0.15	0.24	-0.07	0.45	0.6	1.00					
BIF applications by PMP	0.17	0.26	-0.11	0.12	-0.1	0.14	1.00				
FIP applications by PMP	-0.14	-0.17	0.27	-0.01	-0.1	-0.10	-0.07	1.00			
PERM applications by PMP	0.14	0.22	-0.30	0.07	0.1	0.17	-0.07	-0.04	1.00		
BIF product onsite	0.01	-0.02	-0.01	0.04	-0.1	-0.07	-0.05	-0.03	-0.03	1.00	
PERM product onsite	-0.33	-0.26	0.12	-0.06	0.0	-0.14	-0.11	-0.06	-0.06	0.48	1.00

Supplemental Table 5. Comparison of pesticide measurements in early care and education (ECE) studies. Detection frequencies (DF%) and concentrations (ng/g).

	**UCSF Healthy Children & Environments Study (2017-2019) (n=51)**				**Bradman et al. 2012 (n=39)**				**Morgan et al. 2014 (n=13)**				**Morgan et al. 2007 (n=22)**			
	Location: California, USA				Location: California, USA				Location: North Carolina, USA				Location: Ohio, USA			
**Pesticide Analyte**	DF%	p50	p95	Range	DF%	p50	p95	Range	DF%	p50	p95	Range	DF%	p50	p95	Range
**cis-Permethrin**	98%	234.0	1870.0	<DL-50700	100%	162	940	47-12,712	100%	806	19,700	113–29,000	100%	1010	3,830	127-4,630
**trans-Permethrin**	98%	234.0	1670.0	<DL-56300	100%	225	1501	48-21,058	100%	856	20,900	125–29,900	100%	544	3,420	126-3,950
**Chlorpyrifos**	88%	4.3	18.8	<DL-25.1	92%	11	217	<DL-563	100%	142	921	12.4–921	-	-	-	-
**Cyfluthrin**	10%	<DL	925.0	<DL-1480	5%	<DL	434	<DL-739	42%	<DL	1,750	<DL-1750	-	-	-	-
**Diazinon**	0%	<DL	<DL	<DL	92%	4	61	<DL-74	100%	65.2	6,880	3.06–6,880	-	-	-	-
**Bifenthrin**	94%	151.0	2750.0	<DL-65600	92%	57	897	<DL-928	-	-	-	-	-	-	-	-
**Piperonyl butoxide**	73%	92.7	1160.0	<DL-1770	95%	76	1376	<DL-24,629	-	-	-	-	-	-	-	-
**Cypermethrin**	55%	389.0	3390.0	<DL-12600	41%	<DL	2969	<DL-35,898	-	-	-	-	-	-	-	-
**Dacthal**	6%	<DL	2.9	<DL-3.38	92%	6	51	<DL-74	-	-	-	-	-	-	-	-
**Fipronil**	94%	53.9	412.0	<DL-2430	-	-		-	-	-	-	-	-	-	-	-
**Chlorfenapyr**	33%	<DL	539.0	<DL-1670	-	-		-	-	-	-	-	-	-	-	-
**Deltamethrin**	29%	<DL	1100.0	<DL-14800	-	-		-	-	-	-	-	-	-	-	-
**ƛ-Cyhalothrin**	20%	<DL	236.0	<DL-10400	-	-		-	-	-	-	-	-	-	-	-
**Esfenvalerate**	16%	<DL	324.0	<DL-3940	-	-		-	-	-	-	-	-	-	-	-
